# S100A6 promotes proliferation and migration of HepG2 cells via increased ubiquitin-dependent degradation of p53

**DOI:** 10.1515/med-2020-0101

**Published:** 2020-04-20

**Authors:** Dongqiang Song, Beili Xu, Dongmin Shi, Shuyu Li, Yu Cai

**Affiliations:** Liver Cancer Institute, Department of Hepatic Oncology, Zhongshan Hospital of Fudan University, 180 Fenglin Road, Xuhui District, Shanghai, P. R. China; Department of Gastroenterology and Hepatology, Zhongshan Hospital of Fudan University, 180 Fenglin Road, Xuhui District, Shanghai, P. R. China

**Keywords:** S100A6, hepatocellular carcinoma, ubiquitination, p53, p21

## Abstract

**Purpose:**

S100A6 protein (calcyclin), a small calcium-binding protein of the S100 family, is often upregulated in various types of cancers, including hepatocellular carcinoma (HCC). The aim of this study was to illustrate the molecular mechanism of S100A6 in regulating the proliferation and migration of HCC cells.

**Methods:**

The expressions of S100A6 in human HCC and adjacent non-tumor liver specimens were detected using immunoblotting and quantitative PCR (qPCR). The recombinant glutathione S-transferase (GST)-tagged human S100A6 protein was purified and identified. After treatment with S100A6, the proliferation of HepG2 cells was detected by the MTT and colony formation assay, and the migration of HepG2 cells was investigated by the transwell migration assay; the protein levels of cyclin D1 (CCND1), E-cadherin, and vimentin were also tested by immunoblotting. The effect of S100A6 on p21 and nuclear factor-κB pathway was verified by performing the dual luciferase assay. Then, the expression of p21 and its transcription activator, p53, was examined using immunoblotting and qPCR, the ubiquitination of which was investigated through co-immunoprecipitation.

**Results:**

It was found that the level of S100A6 was higher in the HCC tissues than in the adjacent non-tumor liver specimens. Exogenous overexpression of S100A6 promoted the proliferation and migration of HepG2 cells. S100A6 was observed to regulate p21 mRNA and protein expression levels and decrease p53 protein expression level, not mRNA level, by promoting the ubiquitination of p53 via the proteasome-dependent degradation pathway.

**Conclusion:**

Our study indicated that S100A6 overexpression could promote the proliferation and migration of HCC cells by enhancing p53 ubiquitin-dependent proteasome degradation, ultimately regulating the p21 expression level.

## 1 Introduction

Hepatocellular carcinoma (HCC) contributes to the fourth highest mortality rate of cancer in China, accounting for approximately 40% of the total cases and deaths [1]. Surgical interventions remain the most effective treatment for patients with HCC; however, most cases are diagnosed at an advanced or unresectable stage [2,3]. Although significant improvement has been made in HCC diagnosis and therapy in the past few decades, long-term clinical prognosis and mortality rate are still unsatisfactory [4]; thus, it is imperative that new therapeutic targets merit investigation. This study focused on the pathogenic genes and molecular mechanisms involved in HCC.

S100A6, a member of the S100 calcium-binding protein family, has its gene located at human chromosome 1q21, where chromosomal abnormalities occur frequently [5]. As indicated by the previous studies, S100A6 is associated with tumorigenesis and tumor progression, promoting the proliferation and migration of human colorectal cancer cells, HCC cells, and osteosarcoma cells and epithelial–mesenchymal transition (EMT) of pancreatic cancer cells [6,7,8,9,10]. S100A6 was also found to be associated with the poor prognosis of patients with gastric cancer [11]. S100A6 has been reported to be involved in a number of signaling pathways such as PI3K/Akt, Wnt/β-catenin, p38/MAPK, and nuclear factor (NF)-κB signaling [8,12,13,14].

It is well recognized that p21 acts as a tumor suppressor mainly by inhibiting cell cycle progression and allowing DNA repair [15] and that its role in phenotypic plasticity and its oncogenic function depend on p21 subcellular localization and p53 status [16]. The tumor suppressor p53 is well known to have many anticancer functions, playing a role in apoptosis, genomic stability, and inhibition of angiogenesis [17,18,19,20]. The post-translational modifications are key mechanisms for controlling p53 protein functions [21]. Acetylation, methylation, phosphorylation, neddylation, sumoylation, and ubiquitination all could exert an important downstream effect on the stabilization of p53 and its activation as a transcription factor [21]. The ubiquitination of p53 and functions of the ubiquitin–proteasome pathway (UPP) have been reported to have a more significant impact on p53 protein level and turnover, and more than 20 selective E3 ubiquitin ligases were found to regulate the protein levels and activities of p53 [22].

In this study, we explored the expression of S100A6 in human HCC and adjacent non-tumor liver specimens and investigated the effect of S100A6 protein on the cell proliferation and migration of HepG2 cells, as well as its underlying mechanism involved with p21 and p53.

## 2 Materials and methods

### 2.1 Clinical specimens

As clinical specimens, the six pairs of HCC tissues and adjacent non-tumor tissues were taken from patients with HCC, who underwent liver surgery in the Zhongshan Hospital of Fudan University from 2017 to 2018, with the approval from the Research Ethics Committee of Zhongshan Hospital. The informed consent was obtained from all participants.

### 2.2 Plasmids

The plasmids pGEX4T-1-GST-S100A6, pGEX4T-1-GST, pGL3-NK-κB-luc, and pRL-TK were kindly provided by Prof. Zhaocai Zhou (Chinese Academy of Sciences, Shanghai, China).

### 2.3 Expression and purification of recombinant proteins

The plasmids pGEX4T-1-GST-S100A6 and pGEX4T-1-GST were expressed in the BL21 *Escherichia coli* cells. After isopropyl-beta-D-thiogalactopyranoside (IPTG) (Sangon, China) induction, the cells were pelleted, lysed in phosphate buffered saline (PBS) buffer, and incubated with glutathione beads (GE, USA) to enrich their protein, then the cells were eluted with 25 mM l-glutathione dissolved in PBS buffer and dialyzed in PBS buffer supplemented with 20% glycerol before aliquoted and preserved at −80°C, as previously reported [23].

### 2.4 Cell culture and transfection

The human HCC cell lines HepG2, Hep3B, Huh7, HCCLM3, MHCC97L, and MHCC97H and the normal liver cell line L02 obtained from the Cell Bank of the Chinese Academy of Sciences (Shanghai, China) were cultured in Dulbecco's Modified Eagle Medium (DMEM) supplemented with 10% fetal bovine serum at 37°C under a humidified atmosphere of 5% CO_2_. The plasmids pGL3-p21/NK-κB-luc and pRL-TK were co-transfected into HepG2 cells using Lipofectamine 2000 (Life Technologies, USA) according to the manufacturer’s protocol. The siRNA of S100A6 and random non-coding RNA (GenePharma, China) were transfected into HepG2 cells using Lipofectamine 2000. The efficiency of genetic silencing by the siRNA was evaluated by western blotting.

### 2.5 Cell proliferation assay

HepG2 cells were seeded into a 96-well plate of 2 × 10^3^ cells/each well. After overnight incubation, the cells were treated with different concentrations of GST-S100A6 and GST or not treated; 24, 48, and 72 h later, they were incubated with MTT solution (C0009; Beyotime Biotechnology, China) for 4 h at 37°C, the production of formazan dissolved in dimethyl sulfoxide and quantified spectrophotometrically at a wavelength of 570 nm using a Microplate Reader (Bio-Rad, USA). The experiments were conducted in six replicates and repeated thrice.

### 2.6 Colony formation assay

HepG2 cells were seeded into a six-well-plate of 1 × 10^3^ cells/each well; 2 weeks later, the cells were fixed with 4% paraformaldehyde (Sigma, Germany) and stained with 0.1% crystal violet (C0121; Beyotime Biotechnology, China), then the colony numbers were counted and calculated.

### 2.7 Quantitative reverse transcription PCR

Total RNAs were extracted from the cells using a total RNA kit (Tiangen, China), and complementary DNA was synthesized using ReverTra Ace qPCR RT Master Mix (Toyobo, Japan). Quantitative PCR (qPCR) assay was performed to assess the relative abundances of S100A6, p21, and TP53 mRNAs using specific primers (Table 1), which were stained with SYBR Green (Toyobo, Japan), with an ABI 7500 fast real-time PCR system (ABI, USA). The relative abundances of S100A6, p21, and TP53 were normalized to that of *GAPDH* gene using the ^ΔΔ^Ct method [24]. All data were obtained from three independent experiments.

**Table 1:** Sequences of the primers used in qRT-PCR

Primers for qRT-PCR		
Target gene	Forward primer (5′–3′)	Reverse primer (5′–3′)
*GAPDH*	GAGTCAACGGATTTGGTCGTATTG	ATTTGCCATGGGTGGAATCATATTG
*S100A6*	TCTTCCACAAGTACTCCGGC	CCTTGTTCCGGTCCAAGTCT
*p21*	GCGACTGTGATGCGCTAATG	GAAGGTAGAGCTTGGGCAGG
*TP53*	TGTGACTTGCACGTACTCCC	CTCCGTCATGTGCTGTGACT

### 2.8 Transwell migration assay

The transwell chambers were prepared as 8 mm pores (Corning, USA). HepG2 cells were seeded into the upper chambers of 1 × 10^4^ cells/each well, while the lower chambers were filled with 600 μL medium containing 10% serum, and the cells were supplemented with different concentrations of GST-S100A6 and glutathione S-transferase (GST) or not treated. Then, the cells that migrated to the lower chambers were fixed with 4% paraformaldehyde and stained with 0.5% crystal violet, before counted with an inverted microscope 24 h later.

### 2.9 Immunoprecipitation and immunoblotting

For immunoprecipitation, HepG2 cells were lysed in immunoprecipitation (IP) buffer of 50 mM Tris–HCl, 150 mM NaCl, 5 mM EDTA, 0.1% sodium dodecyl sulfate (SDS), and 1% NP-40 and supplemented with protease inhibitor cocktail, then the cell lysates were incubated with p53 antibody at 1:100 (15104-1-AP; Proteintech, USA) and Protein G agarose beads overnight at 4°C, as previously reported [25]. The immunoprecipitants were enriched and denatured at 100°C for 10 min in 2× SDS-PAGE loading buffer. The inputs, immunoprecipitants, and other cell lysates were then subjected to SDS-PAGE and transferred to the PVDF membrane (Bio-Rad, USA), which were incubated with the appropriate antibodies against S100A6 (1:1,000, 66098-1-Ig; Proteintech, USA), CCND1 (1:1,000, 60186-1-Ig; Proteintech, USA), GST-Tag (1:1,000, 10000-0-AP; Proteintech, USA), ubiquitin (sc-47721; Santa Cruz, USA), p53 antibody (1:2,000, 15104-1-AP; Proteintech, USA), p21 antibody (1:1,000; 60214-1-Ig; Proteintech, USA), E-cadherin (1:1,000, 20874-1-AP; Proteintech, China), vimentin (1:5,000, 10366-1-AP; Proteintech, China), and glyceraldehyde-3-phosphate dehydrogenase (GAPDH) (1:5,000, 60004-1-Ig; Proteintech, USA). The secondary antibodies were labeled with horseradish peroxidase (HRP), and the signals were visualized using Tanon 5200 Imaging System (Tanon, China).

### 2.10 Luciferase reporter assays

HepG2 cells were seeded into 24-well plates of 5 × 10^4^ cells/each well. The cells were cultured overnight and co-transfected with pGL3-p21/NK-κB-luc and pRL-TK plasmids. After 24 h transfection, the cells were treated with GST-S100A6 and GST or not treated. Forty-eight hours later, they were harvested and lysed with 5× passive buffer and subjected to Dual-Luciferase Reporter assay according to the manufacturer’s instruction (E2920; Promega, USA).

### 2.11 Statistical analyses

All experiments were performed in triplicate. All values were depicted as mean ± standard deviation, and the data were analyzed by two-tailed unpaired *t*-tests and one-way ANOVA with Bonferroni *post hoc* test using GraphPad Prism 7. *P < 0.05 was considered to be significant; **P < 0.01 was considered to be more significant.

## 3 Results

### 3.1 Higher expression of S100A6 in human HCC tissues and human HCC cell lines

**Figure 1:**
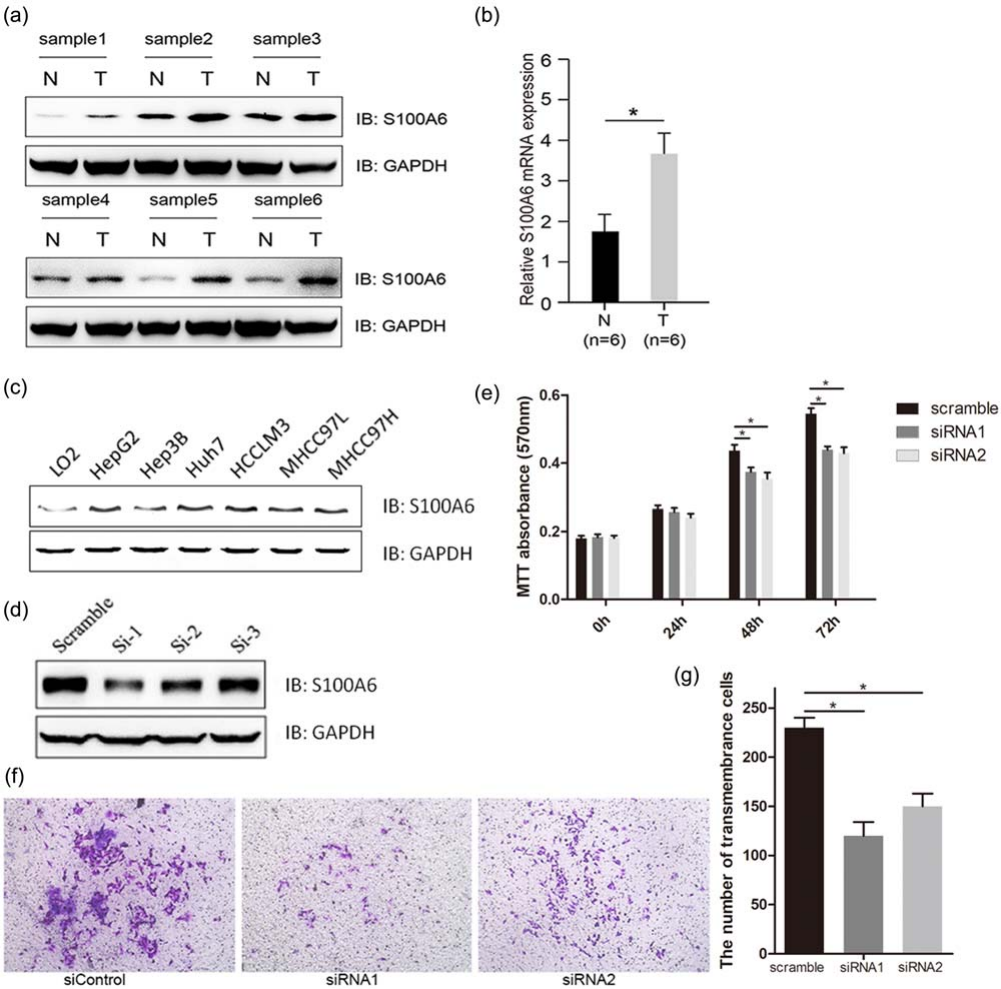
S100A6 expression levels in clinical HCC tissues and adjacent non-tumor tissues. (a) Immunoblotting shows the S100A6 protein levels in different pairs of clinical HCC tissues. (b) Detection of mRNA levels of S100A6 in different pairs of clinical HCC; N: adjacent non-tumor tissue; T: HCC tissues. *P < 0.05. (c) Immunoblotting shows the S100A6 protein levels in different HCC cell lines and the normal liver cell line L02. (d) Immunoblotting shows the S100A6 protein levels in HepG2 cells treated with the siRNA of S100A6 or random non-coding RNA. (e) Detection of the proliferation of HepG2 cells treated with the siRNA of S100A6 or random non-coding RNA by MTT assay; *P < 0.05. (f and g) Detection of the migration of HepG2 cells treated with the siRNA of S100A6 or random non-coding RNA by transwell assay; *P < 0.05.

As indicated in Figure 1a, the immunoblotting, based on the six pairs of the clinical samples, showed that the human HCC tissues presented an upregulation of S100A6 (T) compared with the adjacent non-tumor liver tissues (N). Additionally, the human HCC tissues exhibited a high mRNA level of S100A6 when compared with the adjacent non-tumor liver tissues (Figure 1b). Moreover, the HCC cell lines presented a significant upregulation of S100A6 when compared with the normal liver cell lines (Figure 1c).

### 3.2 Silencing of S100A6 inhibited the proliferation and migration of HepG2 cells

To further investigate the functional roles of S100A6 in HepG2 cells, we first examined the effects of silencing of S100A6 in HepG2 cells. As shown in Figure 1e, S100A6 silencing by siRNA significantly inhibited the growth of HepG2 cells compared with the scrambled control siRNA. Moreover, the downregulation of S100A6 by siRNA significantly decreased the migration in HepG2 cells (Figure 1f and g).

### 3.3 Purification and identification of human S100A6 protein

**Figure 2:**
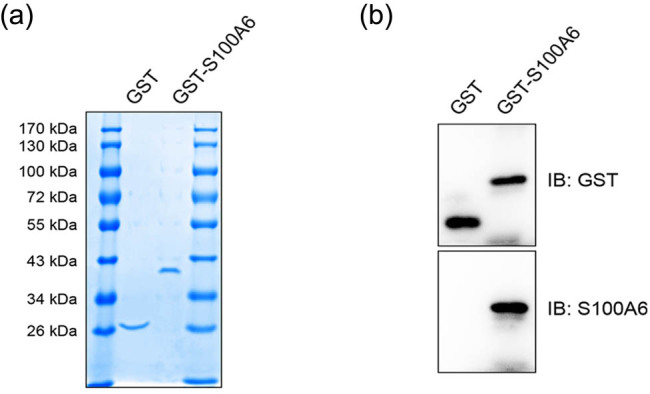
Identification of human GST-S100A6 by Coomassie blue staining and immunoblotting analysis. (a) Detection of recombinant proteins human GST-S100A6 and GST by Coomassie blue staining: GST-S100A6 is approximately 37 kDa and GST is approximately 26 kDa. (b) Detection of recombinant proteins human GST-S100A6 and GST by immunoblotting analysis.

To investigate the effect of extracellular S100A6 on the proliferation and migration of HepG2 cells, recombinant GST-tagged human S100A6 and GST proteins were purified, which was identified by Coomassie blue staining (Figure 2a), and then validated by GST and S100A6 antibodies (Figure 2b). The purified proteins were used to treat the cells in the subsequent experiments.

### 3.4 Exogenous S100A6 promoted the proliferation of HepG2 cells

**Figure 3:**
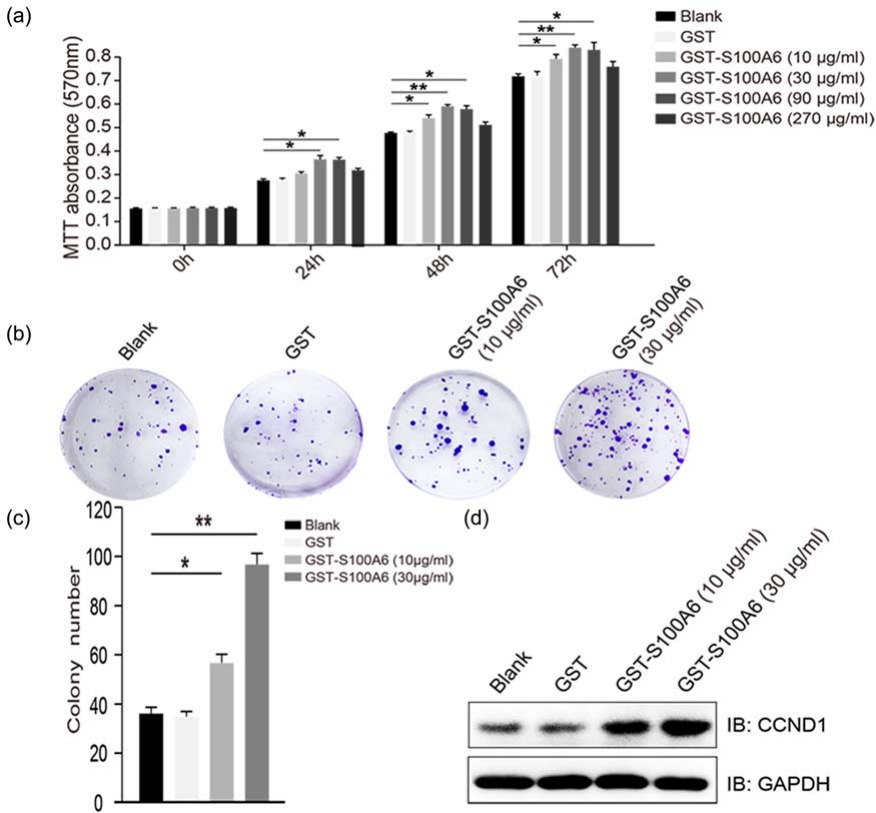
Exogenous S100A6 promotes the proliferation of HepG2 cells. (a) Detection of the proliferation of HepG2 cells by MTT assay; *P < 0.05 and **P < 0.01. (b and c) Colony formation assay of the HepG2 cells treated with GST or GST-S100A6; *P < 0.05 and **P < 0.01. (d) Immunoblotting shows the CCND1 protein levels in HepG2 cells treated with GST or GST-S100A6.

HepG2 cells were treated with GST-S100A6 at the concentrations of 0, 10, 30, 90, and 270 μg/mL for different time periods of 0, 24, 48, and 72 h; their viability was detected by the MTT assay. It was found that GST-S100A6 exhibited a more significant effect on cell proliferation at 30 μg/mL than at other concentrations (Figure 3a). Thus, the concentrations of 10 and 30 μg/mL were selected for all the remaining experiments. Furthermore, after 2-week treatment of GST-S100A6, an increase in the colony number was observed in the GST-S100A6 group (10 and 30 μg/mL) when compared with the blank and GST groups (Figure 3b and c). The protein expression of CCND1, an important marker for cell cycle progression, was elevated after treatment with GST-S100A6 for 48 h (Figure 3d).

### 3.5 Exogenous S100A6 promoted the migration of HepG2 cells

**Figure 4:**
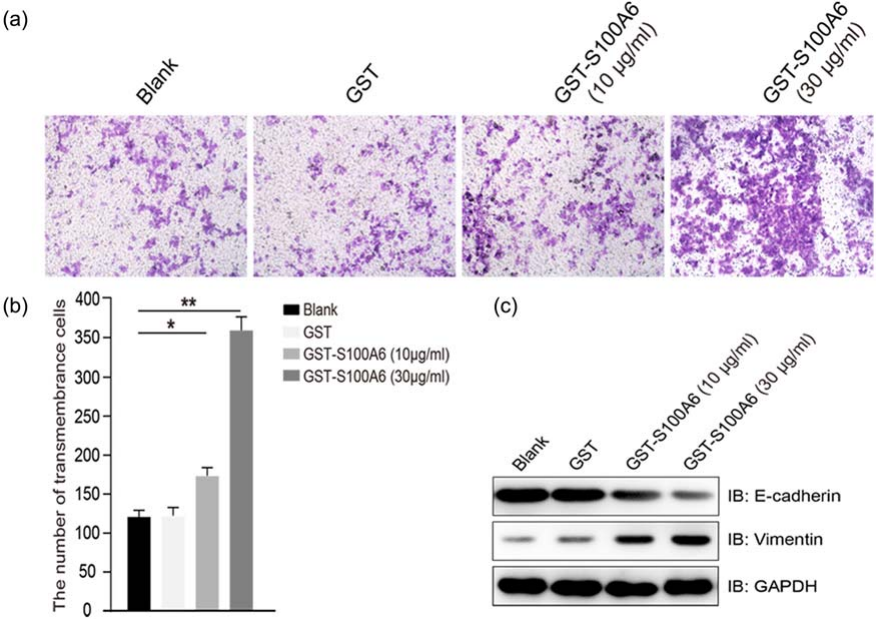
Exogenous S100A6 promotes the migration of HepG2 cells. (a and b) Detection of the migration of HepG2 cells by transwell assay; *P < 0.05 and **P < 0.01. (c) Immunoblotting shows the E-cadherin and vimentin protein levels in HepG2 cells treated with GST or GST-S100A6.

As indicated in Figure 4a and b, the transwell examinations of the effect of S100A6 on the migration of HepG2 cells showed that the number of transmembrane cells was significantly higher in the GST-S100A6 group (10 and 30 μg/mL) than in the blank and GST groups. EMT, one of the important characteristics of tumor metastasis, was found in many tumors in situ. The expression of E-cadherin was decreased and vimentin was increased, the two important markers in the tumor EMT process. Immunoblotting performed to explore the expression of E-cadherin and vimentin after S100A6 treatment in HepG2 cells showed that GST-S100A6 induced a downregulation of E-cadherin and an upregulation of vimentin in a dose-dependent manner (Figure 4c).

### 3.6 Exogenous S100A6 specifically regulated p21 mRNA and protein expression levels

**Figure 5:**
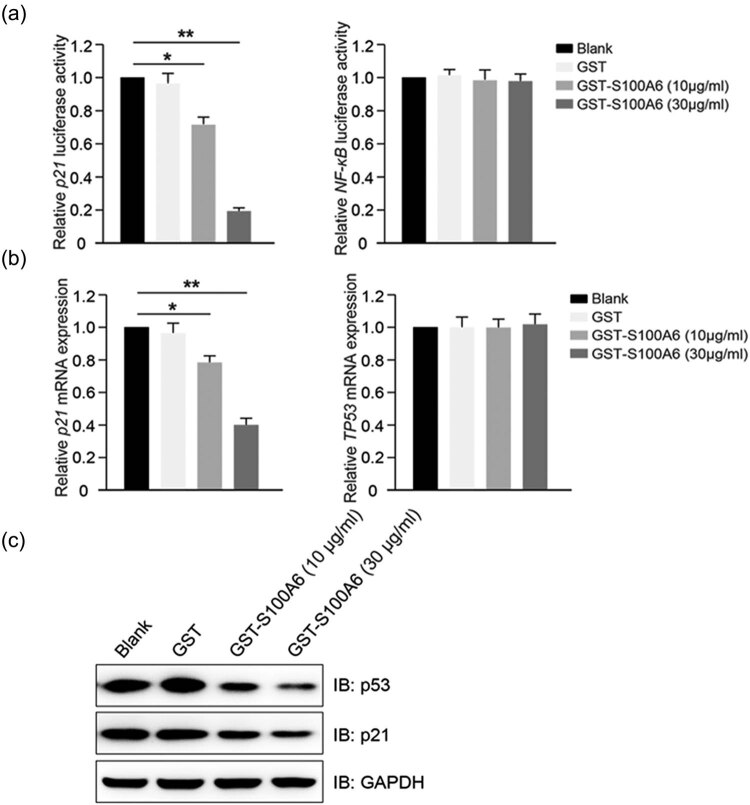
Exogenous S100A6 promotes the degradation of p53. (a) Detection of the p21 and NF-κB luciferase activities of HepG2 cells that treated with GST or GST-S100A6; *P < 0.05 and **P < 0.01. (b) Detection of the p21 and p53 mRNA levels of HepG2 cells treated with GST or GST-S100A6 for 48 h; *P < 0.05 and **P < 0.01. (c) Immunoblotting shows the p21 and p53 protein levels in HepG2 cells treated with GST or GST-S100A6.

Since p21 and NF-κB pathway is well recognized to play an important role in tumor proliferation and migration, we performed dual luciferase reporter experiments to confirm the regulating function of S100A6 in p21 and NF-κB; the results of which indicated that exogenous S100A6 overexpression exhibited a significant inhibitory effect on the p21 luciferase activity but not on the NF-κB luciferase activity (Figure 5a). The mRNA levels of p21 were detected by qPCR; the results showed a significant decrease in p21 mRNA level in HepG2 cells treated with GST-S100A6 compared with that of the blank and GST groups (Figure 5b). Additionally, immunoblotting showed that the p21 protein level decreased with S100A6 overexpression (Figure 5c).

### 3.7 Exogenous S100A6 promoted ubiquitination and degradation of p53

The *p21* gene is known to contain several p53 response elements that mediate direct binding of the p53 protein, resulting in transcriptional activation of the gene encoding the p21 protein. In this study, no significant change was found in p53 mRNA levels (Figure 5b), but the protein levels of p53 were significantly decreased after S100A6 treatment (Figure 5c).

**Figure 6:**
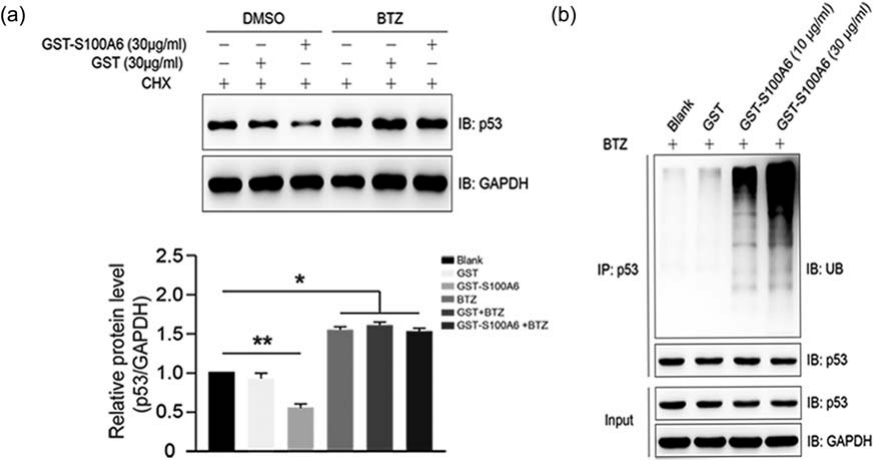
Exogenous S100A6 promotes the ubiquitination of P53. (a) S100A6 promotes the degradation of P53 through proteasome; HepG2 cells were treated with GST/GST-S100A6 and BTZ (bortezomib); the protein levels of p53 normalized to GAPDH; *P < 0.05 and **P < 0.01. (b) S100A6 promotes the ubiquitination of P53; HepG2 cells were treated with GST/GST-S100A6 and BTZ (bortezomib), immunoprecipitated with anti-P53 antibody, and subjected to immunoblotting analysis using the indicated antibodies.

The effect exerted by S100A6 on p53 degradation was rescued by bortezomib (BTZ), a proteasome inhibitor (Figure 6a), which suggested that S100A6 induced p53 degradation through the UPP. Further study indicated that the ubiquitination of p53 was significantly increased in HepG2 cells treated with GST-S100A6 compared with that of the blank and GST groups (Figure 6b).

## 4 Discussion

In this study, we investigated the role of S100A6 in regulating the proliferation and migration of HCC cells through the regulation of p21 and the ubiquitin-dependent degradation of p53. As a result, we found higher mRNA and protein levels of S100A6 in HCC tissues than in the adjacent non-tumor liver tissues. Moreover, we confirmed that S100A6 promotes the proliferation and migration of HepG2 cells. Additionally, we verified the evidence that S100A6 decreased p21 levels and promoted p53 degradation by enhancing its ubiquitin-dependent proteasome pathway.

S100A6, a member of the S100 family, is known to be a calcium (cellular) peripheral protein that regulates cytoskeletal protein dynamics, cell proliferation, differentiation, calcium metabolism, ubiquitination, and acetylation. The S100 protein family consists of 24 members, which are only expressed in vertebrates and show cell-specific expression patterns. Within cells, S100 proteins are involved in the regulation of proliferation, differentiation, apoptosis, Ca^2+^ homeostasis, energy metabolism, inflammation, and migration/invasion through interactions with a variety of target proteins including enzymes, cytoskeletal subunits, receptors, and transcription factors and nucleic acids [26]. It has been reported that increased expression of S100A6 could promote cell proliferation and migration in human HCC [8], which was consistent with the current results that exogenous S100A6 promotes the proliferation and migration of HepG2 cells. A number of previous studies have reported that EMT has become a principal factor in tumor malignancy, as EMT contributes to the motility and invasiveness of tumor cells, thereby leading to distant metastasis [27,28]. The current results confirmed that S100A6 induced EMT in HepG2 cells with decreased E-cadherin and increased vimentin expressions.

Of note, S100A6 at the higher concentrations (90 and 270 μg/mL) did not show more significant effect on the proliferation of HepG2 cells compared with that at the lower concentration (30 μg/mL), which was consistent with other members of the S100 family, such as S100A9 [29], suggesting that the S100 protein family can exert its biological functions in a concentration-dependent manner.

S100A6 has been reported to be involved in a number of signaling pathways, such as PI3K/Akt, Wnt/β-catenin, p38/MAPK, and NF-κB signaling [8]. This also suggested that S100A6 might play a role in DNA damage repair, revealing another potential carcinogenic mechanism [30]. Tumor necrosis factor-α (TNF-α) could induce the gene expression of S100A6 through the NF-κB pathway in HepG2 cells [14]. In this study, exogenous S100A6 had no effect on the NF-κB luciferase activity. When we targeted p21, however, we found that S100A6 could decrease both mRNA and protein levels of p21.

It was reported that together with p53, p21 could constitute the cell cycle G1 checkpoint, and p53 could inhibit the progression of the cell cycle by inducing p21 [31], which lead us to examine the levels of p53, a transcription activator of p21, after the overexpression of S100A6. As a tumor suppressor, p53 could play an important role in G1/S transition and growth arrest in the cell cycle [32]. The overexpression of S100A6 could promote the degradation of p53 acetylation, thus leading to the production of cancerous cells [32]. Moreover, the ubiquitination of p53 could have a significant impact on p53 protein levels and turnover [20]. In this study, we found that S100A6 promoted the degradation of p53 through the enhancement of ubiquitination, and meanwhile, we did not observe any changes in p53 mRNA levels after the overexpression of S100A6.

Therefore, we assume that S100A6 can be an important marker for HCC. Efforts are needed in our future studies to investigate the functions of S100A6 in animal models and more patient samples.

## 5 Conclusion

Our research suggests that S100A6 could promote the proliferation and migration of HCC by increasing the degradation and ubiquitination of p53, which reveals a new mechanism of S100A6 in HCC development, thus offering a potent therapeutic target for HCC treatment.
